# Diesel exhaust but not ozone increases fraction of exhaled nitric oxide in a randomized controlled experimental exposure study of healthy human subjects

**DOI:** 10.1186/1476-069X-12-36

**Published:** 2013-04-20

**Authors:** Stefan Barath, Nicholas L Mills, Ellinor Ädelroth, Anna-Carin Olin, Anders Blomberg

**Affiliations:** 1 Department of Public Health and Clinical Medicine, Division of Medicine, Umeå University, Umeå, Sweden; 2 BHF/University Centre for Cardiovascular Science, Edinburgh University, Edinburgh, UK; 3 Department of Occupational and Environmental Medicine, Sahlgrenska University Hospital, Gothenburg, Sweden

**Keywords:** Air pollution, Particulate matter pollution, Airway inflammation

## Abstract

**Background:**

Fraction of exhaled nitric oxide (FENO) is a promising non-invasive index of airway inflammation that may be used to assess respiratory effects of air pollution. We evaluated FENO as a measure of airway inflammation after controlled exposure to diesel exhaust or ozone.

**Methods:**

Healthy volunteers were exposed to either diesel exhaust (particle concentration 300 μg/m^3^) and filtered air for one hour, or ozone (300 ppb) and filtered air for 75 minutes. FENO was measured in duplicate at expiratory flow rates of 10, 50, 100 and 270 mL/s before, 6 and 24 hours after each exposure.

**Results:**

Exposure to diesel exhaust increased FENO at 6 hours compared with air at expiratory flow rates of 10 mL/s (p = 0.01) and at 50 mL/s (p = 0.011), but FENO did not differ significantly at higher flow rates. Increases in FENO following diesel exhaust were attenuated at 24 hours. Ozone did not affect FENO at any flow rate or time point.

**Conclusions:**

Exposure to diesel exhaust, but not ozone, increased FENO concentrations in healthy subjects. Differences in the induction of airway inflammation may explain divergent responses to diesel exhaust and ozone, with implications for the use of FENO as an index of exposure to air pollution.

## Background

Air pollution is recognized as a major health problem worldwide with exposure to air pollutants responsible for adverse health effects and, in particular, increases in cardio-respiratory morbidity and mortality [1]. Both diesel exhaust (DE) and ozone (O_3_) are important urban air pollutants and share some toxicological mechanisms through oxidative stress and airway inflammation [2,3].

Both epidemiological and toxicological research supports a link between urban air pollution and an increased incidence and severity of airway disease. Detrimental effects of ozone and particulate matter (PM) on respiratory symptoms and function are well documented. There is not only strong epidemiological evidence of a relationship between air pollution and exacerbation of asthma and respiratory morbidity and mortality in patients with chronic obstructive pulmonary disease (COPD), but recent studies have also suggested a role for pollutants in the development of both asthma and COPD [4].

Short-term increases in PM levels are associated with an increased risk of cardiopulmonary mortality [5]. Ozone has been shown to cause decreases in lung function and has been associated with various respiratory symptoms including dyspnea, upper airway irritation, coughing and chest tightness [6]. Whilst a significant increase in the risk of death from respiratory causes has been demonstrated in association with increases in ozone concentrations, the effect of ozone on cardiovascular mortality is less clear [7].

In healthy subjects, controlled exposures to diesel exhaust at a PM concentration of 300 μg/m^3^ result in mucosal inflammation of the airways with significant increases in neutrophils, lymphocytes and mast cells along with upregulation of the vascular endothelial adhesion molecules intercellular adhesion molecule 1 (ICAM-1), vascular cell adhesion protein 1 (VCAM-1) and P-selectin as early as 6 hours after exposure. Increases in inflammatory markers have also been pronounced in bronchial wash, whereas signs of airway inflammation in the more distally sampling bronchoalveolar lavage have been modest [8-11]. This airway inflammatory response is mediated through increased expression of the important oxidative stress-sensitive transcription factors nuclear factor kappa b (NFkB) and activator protein 1 (AP-1) as well as mitogen activated protein kinases (MAPkinases) (9). Exposure to ozone also induces airway inflammation with increases in interleukin (IL)-6, IL-8, granulocyte-macrophage-colony-stimulating factor (GM-CSF) and prostaglandin E_2_ identified in bronchoalveolar lavage fluid, along with a neutrophil recruitment to the airway mucosa, bronchial wash and bronchoalveolar lavage [12-14]. However, in contrast to the marked changes in epithelial cell transcription induced by exposure to diesel exhaust, ozone does not alter NFκB expression in the airway epithelium [12].

Understanding the mechanisms through which exposure to air pollutants influence airway inflammation in health and disease is an important first step in the attempts to reduce the impact of air pollution on human health. However, invasive studies involving bronchoscopy and biopsy do not lend themselves to assessment of the effects of air pollution at ambient concentrations in real world settings and, therefore, there is a need to identify simple non-invasive methods for assessing the effects of air pollution on airway inflammation.

In recent years, the fraction of exhaled nitric oxide (FENO) has been employed as a non-invasive index of allergic and eosinophilic airway inflammation. The concentration of FENO is inversely related to flow-rate. Thus, when exhaling at lower flow rates, more NO is contributed from the central airways relative to the overall concentration in the breath. This characteristic pattern occurs because the slower flow rate allows more time for NO to enter from the airway and be exhaled. Based on this, measuring FENO at multiple expiratory flow rates may be used as a simple means to reflect inflammation at different levels of the airway tract [15,16].

FENO has been used to evaluate the effect of air pollution in children with asthma [5] and Steerenberg et al. report an association between FENO and levels of nitrogen dioxide (NO_2_), carbon monoxide (CO), PM_2.5_ and pollen in children [17,18]. The effects of individual pollutants on FENO are difficult to determine in observational studies and, thus, controlled experimental studies are necessary to evaluate the potential of FENO as an index of exposure to atmospheric pollutants.

We hypothesized that exposure to diesel exhaust or ozone, common and highly oxidative air pollutants, would increase FENO and that measurements of FENO at multiple flow rates and time points would reflect differences in the distribution and induction of airway inflammation associated with these pollutants.

## Methods

### Subjects

Thirty-six healthy male volunteers participated in the study (Table 1). The volunteers were non-smokers with no history of asthma or allergy, and had a normal physical examination, normal spirometry and negative skin prick tests to ten common aeroallergens. The study was performed following approval by the local Ethics Review Board, in accordance with the Declaration of Helsinki, and with written informed consent from all volunteers.

**Table 1:** Baseline demographics of healthy male volunteers

	**Diesel exhaust**	**Ozone (A)**	**Ozone (B)**
Number (n)	10	12	18
Age (years)	26 ± 2	26 ± 2	26 ± 3
Height (cm)	179 ± 6	180 ± 6	183 ± 8
Weight (kg)	78 ± 9	78 ± 9	78 ± 9
Body Mass Index	24 ± 3	24 ± 2	23 ± 3
Lung function			
FVC (L)	6.0 ± 0.6	5.9 ± 0.6	5.8 ± 0.9
FEV_1 _(L)	4.3 ± 0.3	4.2 ± 0.5	4.4 ± 0.6

Data are reported as mean ± SD.

FEV_1_ = Forced expiratory volume in one second.

FVC = Forced vital capacity.

### Subject preparation

All individuals were asked to refrain from alcohol containing beverages for 24 hours and from coffee 4 hours prior to the exposure. Subjects were fasted from midnight and provided with a standardized nitrate-low diet following exposure.

### Study design

Using a double blind cross-over design, subjects were exposed to either diesel exhaust and filtered air for 1 hour (n = 18) or ozone and filtered air (n = 18) for 75 minutes. The exposures were carried out in randomized order and were separated by at least two weeks. A second cohort of 18 healthy volunteers were exposed to ozone and filtered air for 75 minutes using an identical study design giving a final study population of 36 for the comparison between ozone and filtered air. During exposures, subjects performed light exercise on a bicycle ergometer alternated with rest at 15-minute intervals. The bicycle workload was standardized to achieve a minute ventilation of 20 L/min/m^2^ body surface area.

### Exposures

All exposures were performed in the morning in two separate, purpose-built, chambers for studying the effects of ozone and diesel exhaust respectively, as previously described [8,13]. Diesel exhaust was generated by a diesel engine from 1991 (Volvo TD40 GJE, 4.0 L, four cylinders) connected to an engine dynamometer and running under control of a computer program according to the European Transient Cycle (ETC.), as previously reported [19]. The fuel used was Statoil class 1 diesel fuel. A Tapered Element Oscillating Microbalance (TEOM 1400) instrument was used as well as a standard glass fiber filter for monitoring the PM_10_ levels in the chamber. The PM_10_ mass concentration was approximately 300 μg/m^3^. To obtain this, a partial flow of DE was drawn and then diluted with filtered air and fed into the chamber, as previously described in detail [8].

Ozone was generated by a Fischer’s O_3_ generator 500 MM (Fischer Labor and Verfahrens-Technik, Bonn, Germany). The chamber concentration was continuously monitored photometrically by an ozone analyser (Dasibi model 1108, Dasibi Environmental Corp., California, USA) and maintained at 300 ppb. During the ozone exposure, ambient air was continuously drawn through the exposure chamber to maintain a temperature of 20°C and a relative humidity level of 50% (13). Exposure to filtered air was performed in the same facilities and with the same environmental setting as the ozone or DE exposure. All exposures were blind to the investigator and the subjects and known only by the technical staff.

### Lung function assessments

Dynamic spirometry variables (FVC and FEV_1_) were determined pre- and post-exposure (2 hours) using a conventional spirometer (Vitalograph, Buckingham, UK).

### Fraction of exhaled nitric oxide (FENO)

Volunteers had a nose clip applied before being asked to inhale nitrogen oxide free air and then exhale slowly against a resistance according to ATS/ERS recommendations [20]. FENO with flow rates between 10 and 270 mL/s (FENO_10_, FENO_50_, FENO_100_ and FENO_270_) were measured in duplicate before, and 6 and 24 hours after the end of each exposure using a chemiluminescence analyser (Ni_OX_, Aerocrine AB, Stockholm, Sweden). The research nurses responsible for the FENO measurements were blind to the actual exposure.

### Statistical analyses

Data are presented as mean ± SD. A repeated measures analysis of variance (General Linear model) with two within-subject factors (time and exposure) was used, with pre-exposure FENO data used as a reference using SPSS, version 16.0 (SPSS Inc., Chicago, IL, USA). In order to avoid type-I errors due to two comparisons, the level of significance was adjusted by dividing the set significance level by two (Bonferroni correction) and therefore statistical significance was taken at p < 0.025.

## Results

There were no significant differences in the age or demographics of the healthy volunteers exposed to diesel exhaust or ozone (Table 1). FENO data from 8 subjects exposed to diesel exhaust or filtered air as well as data from 6 subjects exposed to ozone or filtered air in the first ozone cohort were not suitable for analysis due to instrument failure. This gives a final study population of n = 10 for diesel exhaust. Collection of FENO data was complete for the second cohort of volunteers exposed to ozone and filtered air and results from the two ozone-exposed cohorts were combined to give a final study population of n = 30.

**Figure 1:**
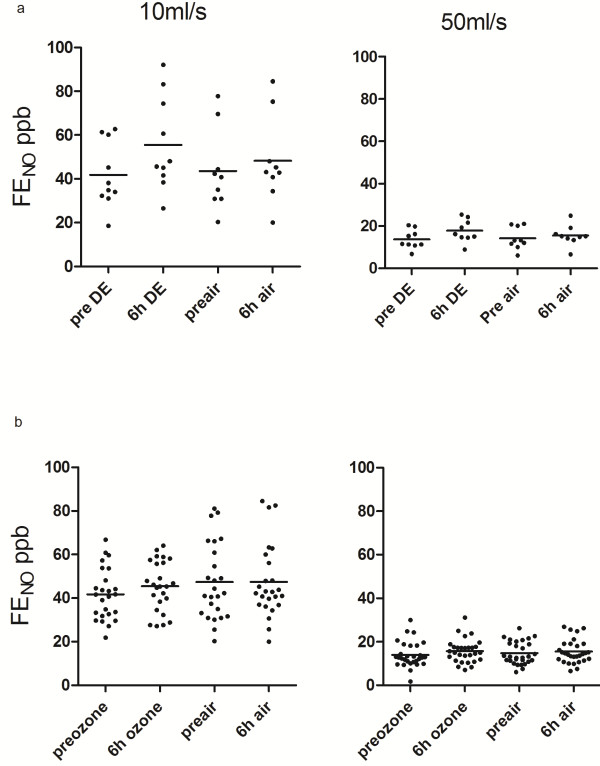
**FENO responses after exposure to diesel exhaust and ozone. a) **Exposure to diesel exhaust for one hour increased the fraction of exhaled nitric oxide (FENO) compared to filtered air at expiratory flow rates of FENO_10 _[60.8 ± 6.0 *vs*. 50.2 ± 5.9 ppb repeated measures ANOVA P = 0.01] and at FENO_50 _[18.6 ± 1.6 *vs*.15.9 ± 1.5 ppb repeated measures ANOVA P = 0.011]. **b) **Exposure to ozone for one hour did not alter FENO concentrations compared to filtered air at expiratory flow rates of FENO_10 _and FENO_50_.

Exposure to diesel exhaust or ozone did not affect lung function compared to filtered air (Table 2). There were no significant differences in baseline FENO concentrations between exposures (Table 3 and Figure 1a). Exposure to diesel exhaust for one hour increased FENO at 6 hours compared to filtered air at expiratory flow rates of 10 mL/s [58.8 ± 21.0 ppb *versus* 49.9 ± 18.8 ppb; p = 0.01] and at 50 mL/s [17.7 ± 5.6 ppb *versus* 15.7 ± 4.8 ppb; p = 0.011] (Figure 1a), but FENO concentrations returned to baseline by 24 hours. FENO concentrations were not significantly affected by diesel exhaust exposure compared to filtered air at higher flow rates (Table 3 and Figure 1b).

**Table 2:** Lung function following exposure to diesel exhaust or ozone

	**Diesel exhaust**	**Filtered air**	**Ozone**	**Filtered air**
	**(n = 10)**	**(n = 10)**	**(n = 30)**	**(n = 30)**
*FEV*_*1*_*, L*				
Pre-exposure	4.3 ± 0.31	4.3 ± 0.31	4.3 ± 0.5	4.3 ± 0.5
2 hours	4.3 ± 0.63	4.3 ± 0.31	4.2 ± 0.6	4.3 ± 0.5
*FVC, L*				
Pre-exposure	6.1 ± 0.63	6.0 ± 0.63	5.8 ± 0.8	5.8 ± 0.8
2 hours	6.1 ± 0.63	6.1 ± 0.95	5.6 ± 0.8	5.7 ± 0.8

Data are reported as mean ± SD.

FEV_1_ = Forced expiratory volume in one second.

FVC = Forced vital capacity.

**Table 3:** Fraction of exhaled nitric oxide (FENO ppb) in healthy volunteers exposure to diesel exhaust or ozone

***Flow rate***	**FENO**_**270**_	**FENO**_**100**_	**FENO**_**50**_	**FENO**_**10**_	**FENO**_**270**_	**FENO**_**100**_	**FENO**_**50**_	**FENO**_**10**_
	**Air**				**Diesel**			
Pre exposure	3.9 ± 1.5	8.4 ± 3.1	14.5 ± 5.3	46.6 ± 18.5	4.0 ± 1.0	8.2 ± 2.5	14.0 ± 4.7	46.2 ± 16.8
6 hours	4.1 ± 1.4	9.9 ± 2.7	15.7 ± 4.8	49.9 ± 18.8	4.7 ± 1.7	10.5 ± 3.7	17.7 ± 5.6#	58.8 ± 21.0*
24 hours	4.0 ± 1.7	9.0 ± 3.1	14.6 ± 5.4	47.2 ± 20.9	4.4 ± 1.0	8.8 ± 2.6	15.3 ± 4.5	50.2 ± 15.6
	**Air**				**Ozone**			
Pre exposure	4.6 ± 1.7	9.3 ± 4.0	16.1 ± 7.5	57.3 ± 31.6	4.5 ± 1.5	8.5 ± 2.7	14.1 ± 6.1	49.0 ± 20.1
6 hours	4.7 ± 1.8	10.0 ± 4.6	17.1 ± 8.7	58.3 ± 35.8	4.6 ± 1.4	9.1 ± 2.8	15.7 ± 5.3	51.0 ± 21.3
24 hours	4.8 ± 1.9	9.9 ± 4.4	16.7 ± 8.1	59.1 ± 32.7	4.4 ± 1.2	8.9 ± 2.8	14.7 ± 4.5	50.1 ± 17.8

Diesel exhaust versus air: #p = 0.011 *p = 0.010.

Values are reported as mean ± SD.

Exposure to ozone did not affect FENO at any flow rate or time point in either cohort of healthy volunteers. FENO concentrations following exposure to ozone and filtered air in all subjects are reported in Table 3.

## Discussion

As a non-invasive index of airway inflammation, FENO has not previously been studied following exposure to diesel exhaust or, at multiple flow rates, after ozone exposure. Here, FENO was measured after controlled exposure to either diesel exhaust or ozone at levels previously reported to induce airway inflammation. As more NO is contributed from the central airways relative to the overall concentration in the breath, when exhaling at lower flow rates, FENO concentrations obtained using different flow rates may be suggested to reflect different parts of the bronchial tree. Thus lower flow rates (FENO_10_-FENO_50_ mL/s) correspond to the central airways and the highest flow rate (FENO_270_ mL/s) to the distal airways [15,16].

Exposure to diesel exhaust for one hour increased FENO in healthy subjects at 6 hours. However, only FENO concentrations obtained at lower exhalation flow rates (FENO_10_ and FENO_50_) were affected, suggesting that the central airways are mainly involved. This observation is in concordance with previous studies, in which diesel exhaust-induced increases in inflammatory cells and cytokines have been identified in specimens obtained from the central airways, mucosal biopsies and bronchial washings, but not from bronchoalveolar lavage [8-11]. Increases in FENO_10_ and FENO_50_ concentrations were transient and had returned to pre-exposure levels at 24 hours.

In contrast, exposure to ozone did not affect FENO at any flow rate or any time point. To increase the power of the study to confirm a true negative response or to detect a small effect of ozone on FENO, we repeated the study, by doubling the number of subjects from 18 to 36. However, no effect of ozone on FENO was found in either cohort or in the combined data set, suggesting that ozone-induced acute airway inflammation is not possible to detect using FENO. These findings are consistent with three previous studies in healthy subjects, implying that experimental exposure to ozone does not affect FENO [21-23]. The lack of an ozone-induced increase in FENO in previous human experimental exposure studies has been suggested to be due to the relatively low dose of ozone employed (0.2 ppm) or that, whilst ozone in fact may increase the production of NO within the airways, the ozone-induced neutrophilic airway inflammation would lead to the production of superoxide that reacts with NO, masking any increase in exhaled NO.

The present findings from controlled ozone exposures are in contrast to the positive associations reported between exposure to ozone and FENO in previous field studies [24]. It is possible that the effects of FENO suggested to be related to ozone exposure only occur following repeated exposure or in the presence of other ambient pollutants. Alternatively, observational studies may suffer from residual confounding factors and may incorrectly attribute fluctuations in FENO to ozone exposure, as there is a complex relationship between ozone and other pro-inflammatory pollutants such as PM_10_.

Both diesel exhaust and ozone are considered oxidant air pollutants and exert their effects on the airways through oxidative stress [3,25,26]. The different effects on FENO by the two exposures could be explained by the fact that the ozone molecule is highly reactive and therefore does not reach the airway epithelium but reacts with molecules within the respiratory tract lining fluid to cause a cascade of secondary free radical-derived ozonation products [3,25]. In contrast, DEPs are deposited on the airway epithelium, where they induce a local inflammatory response and may also translocate to affect the local vascular endothelium [26,27].

Exhaled NO production is thought to be under the regulation of three endothelial NOS (nitric oxide synthase) isoforms. NOS I and II are predominantly expressed in healthy subjects, whilst NOS III is up-regulated in patients with asthma. Recently, there is evidence that exhaled NO is associated with a genetic variant of NOS III in patients with asthma, suggesting both NOS II and NOS III to be important in determining the exhaled NO in this patient group [28]. Endothelial nitric oxide synthetase (NOS III) is regulated under the influence of the oxidative stress-sensitive transcription factor NFκB. NFκB activation and upregulation of NOS III occur in endothelial cells exposed to reactive oxygen species [29,30]. We have previously demonstrated that NFκB, along with AP-1 and MAPkinases, are activated by exposure to diesel exhaust [9], and it is plausible that this may in turn lead to the upregulation of NOS III. In contrast, exposure to ozone has not been found to activate NFκB in human airways, which suggests a difference in the induction of the airway inflammatory response between ozone and DE [12]. Taken together, these observations suggest that ozone exposure may not influence FENO because it does not, in isolation, activate NFκB and upregulate NOS III.

In a study by Mehta *et al.,* levels of exhaled NO were increased following infusion of the NO precursor L- Arginine, indicating that exhaled NO reflects endogenous production of NO [31]. Interestingly, basal concentrations and changes in exhaled NO in that study were similar to the increase in FENO following exposure to diesel exhaust in the present study. Previously, we have hypothesized that the DE-induced oxidative stress and the subsequent adverse cardiovascular health effects are mediated through reduced NO bioavailability [32]. Oxidative stress caused by exposure to DE and subsequent consumption of vascular NO may evoke homeostatic mechanisms to normalize vascular function through the upregulation of NOS III, which in turn may increase FENO. It can thus be speculated upon that the effect on FENO detected following DE exposure rather is related to a vascular response than to airway inflammation. However, previous DE exposure studies have revealed that the time-kinetics of the vascular and airway responses are quite similar with a peak response around six hours after exposure [8,32].

There are a number of potential limitations that merit discussion. Firstly, this study has been conducted in healthy individuals and we know from previous studies that patients with asthma show a different airway inflammatory response when exposed to diesel exhaust compared with healthy individuals [11]. The number of subjects, in whom data were available from exposures to diesel exhaust, was small and it is possible that the study was underpowered to exclude an effect of DE exposure on FENO_100_ and FENO_270_. Furthermore, whilst the DE-induced FENO changes seen at the 10 and 50 mL/s flow rates were clearly significant, it cannot be fully excluded, however less probable, that these changes were due to chance alone. Airway inflammation was not addressed invasively in the present study and, thus, no correlation analyses between FENO and airway inflammation were possible.

## Conclusions

Exposure to diesel exhaust, but not ozone, increases the concentration of FENO_10_ and FENO_50_ in healthy subjects, suggesting an inflammatory response mainly located in the central airways. This is consistent with previous invasive studies that identify an established airway inflammation in bronchial wash and endobronchial mucosal biopsies following exposure to diesel exhaust. The divergence in response to diesel exhaust and ozone may be found in differences in NFκB activation or as a consequence of different vascular endothelial responses, but the precise mechanism whereby exposure to PM increases FENO requires further research. Our observations support the use of FENO at multiple expiratory flow rates as a non-invasive means to assess the inflammatory response in different parts of the bronchial tree.

## Abbreviations

AP-1: Activator protein 1; CO: Carbon monoxide; COPD: Chronic obstructive pulmonary disease; DE: Diesel exhaust; ETC.: European Transient Cycle; FENO: Fraction of exhaled nitric oxide; FEV1: Forced exhaled volume in 1 second; FVC: Forced vital capacity; GM-CSF: Granulocyte-macrophage-colony-stimulating factor; ICAM-1: Intercellular adhesion molecule 1; IL: Interleukin; MAPkinases: Mitogen activated protein kinases; NFkB: Nuclear factor kappa b; NO: Nitric oxide; NO2: Nitrogen dioxide; NOS: Nitric oxide synthase; O3: Ozone; PM: Particulate matter; PM2.5 /PM10: Particulate matter with a diameter of less than 2.5 and 10 μm respectively; ppb: Parts per billion; VCAM-1: Vascular cell adhesion protein 1.

## Competing interests

The authors have no financial or non-financial competing interests to declare.

## Authors’ contributions

SB took part in study design, was responsible for coordinating the study, performed the data analysis and drafted manuscript. NM and A-CO participated in study design, interpretation of data and manuscript preparation. EÄ helped to draft and revise the manuscript. AB was included in study design, carried out statistical analyses and took part in manuscript preparation. All authors read and approved the final manuscript.
